# Construction and validation of a prognostic nomogram for ductal adenocarcinoma of the prostate: A population-based study

**DOI:** 10.1097/MD.0000000000036877

**Published:** 2024-01-12

**Authors:** Cheng Li, Zhengqiang Wan, Yinglei Wang, Guangming Shan, Baoquan Yang

**Affiliations:** a The Second Clinical Medical College of Binzhou Medical University, Yantai, Shandong, China; b The Second Ward of Urology, Yantai Affiliated Hospital of Binzhou Medical University, Yantai, Shandong, China.

**Keywords:** DAC, nomogram, overall survival, prognosis, SEER

## Abstract

This study aimed to establish and validate a nomogram for ductal adenocarcinoma of the prostate (DAC) to accurately predict the prognosis of DAC patients. The data of 834 patients with confirmed DAC were obtained from the Surveillance, Epidemiology, and End Results database. The cases were randomly assigned to the training and internal validation cohorts. Data from patients attending our institution as an external validation cohort (n = 35). Nomogram and web-based dynamic nomogram were constructed based on Cox regression analysis, and their prediction accuracy was evaluated by concordance index (C-index), calibration curve, receiver operating characteristic (ROC) curve, and decision curve analysis. Multivariate analyses identified age, T-stage, N-stage, M-stage, surgery, lymph node dissection, Gleason score, and PSA as independent prognostic factors for overall survival. The C-index and calibration curves demonstrate the good discriminative performance of the prediction model. The area under the curve further confirmed the accuracy of the nomogram in predicting survival. In addition, the area under the curve and decision curve analysis were better than the 7th tumor-node-metastasis staging system. The Kaplan–Meier curves of the nomogram-based risk groups showed significant differences (*P* < .001). We constructed and validated the first nomogram to predict patients with DAC.

## 1. Introduction

Prostate cancer (PCa) is one of the most common malignancies and the 5th leading cause of cancer deaths in men worldwide. In 2020, there were 1414,249 new diagnoses and 375,000 deaths annually worldwide.^[1,2]^ The pathogenesis of PCa is currently unknown, but studies suggest a strong genetic link, while race, obesity, persistently elevated testosterone levels, and high blood pressure increase the risk of developing PCa.^[3,4]^ In 2016, the World Health Organization classification of tumors of the urinary system and male genital organs identified rare histological sub-types of PCa other than the most common acinar adenocarcinoma (APC), including neuroendocrine tumors, basal cell carcinoma, adenosquamous carcinoma and ductal adenocarcinoma of the prostate (DAC).^[5]^ Of these, DAC is a rare sub-type of PCa, with an incidence of <1%, but remains the second most common sub-type of PCa.^[6]^ DAC was developed by Melicow in 1967 and was initially referred to as “endometrioid carcinoma” or “papillary carcinoma” because of its early pathological resemblance to endometrial cancer.^[7]^ The prognosis is often poor due to the lack of specific treatment.^[8]^ DAC is also more likely to metastasize to less common sites than APC, including the lung, brain and testis.^[9,10]^ The clinical presentation of DAC is mostly hematuria, which lacks specific clinical features. At the time of diagnosis, prostate-specific antigen (PSA) levels are usually low in patients with DAC.^[11,12]^ Decision curve analysis (DCA) is difficult to diagnose in the early stages of the disease and can be easily missed or even misdiagnosed, with most diagnoses being made at a late stage, thus missing the best time for treatment and resulting in a poor prognosis.

Due to the rarity of DAC, studies on the clinical features and prognosis of DAC are scarce and limited to a few case reports and small retrospective studies^[13–15]^ and the current divergent views on its biology, prognosis and outcome limit the choice of optimal treatment strategies. The most commonly used method for predicting the prognosis of patients with DAC is the American Joint Committee on Cancer (AJCC) tumor-node-metastasis (TNM) system, which does not take into account factors such as age, race, pathological stages and treatment options that may have an impact on patient prognosis. We aim to develop nomogram for patients with DAC and to explore the factors that influence their survival prognosis.

## 2. Materials and methods

### 2.1. Data sources and study subjects

In this retrospective cohort study, we extracted clinical information about patients diagnosed with primary DAC by accessing the Surveillance, Epidemiology, and End Result (SEER) database. We used the International Classification of Diseases in Oncology (ICD) and ICD-O-3 morphology codes to differentiate patients with a diagnosis of DAC. In addition, as the relevant TNM stages diagnosis was missing for patients before 2004, to ensure that the selected cases had complete follow-up time and survival outcomes, we selected patients diagnosed with ductal adenocarcinoma of the prostate between 2004 and 2017. Variables for each patient included age, race, marital status, AJCC TNM stages, surgery, PSA, chemotherapy, radiotherapy, lymph node dissection (LND), tumor grading and preoperative Gleason score. The AJCC TNM stages (7th edition) was used for the analysis. In addition, for the survival analysis we collected time to survival (months), cause of death of patients. The endpoint of this study was overall survival (OS). OS was defined as the duration from the time of diagnosis and death or the final follow-up.

### 2.2. Inclusion and exclusion criteria

**Figure 1.**
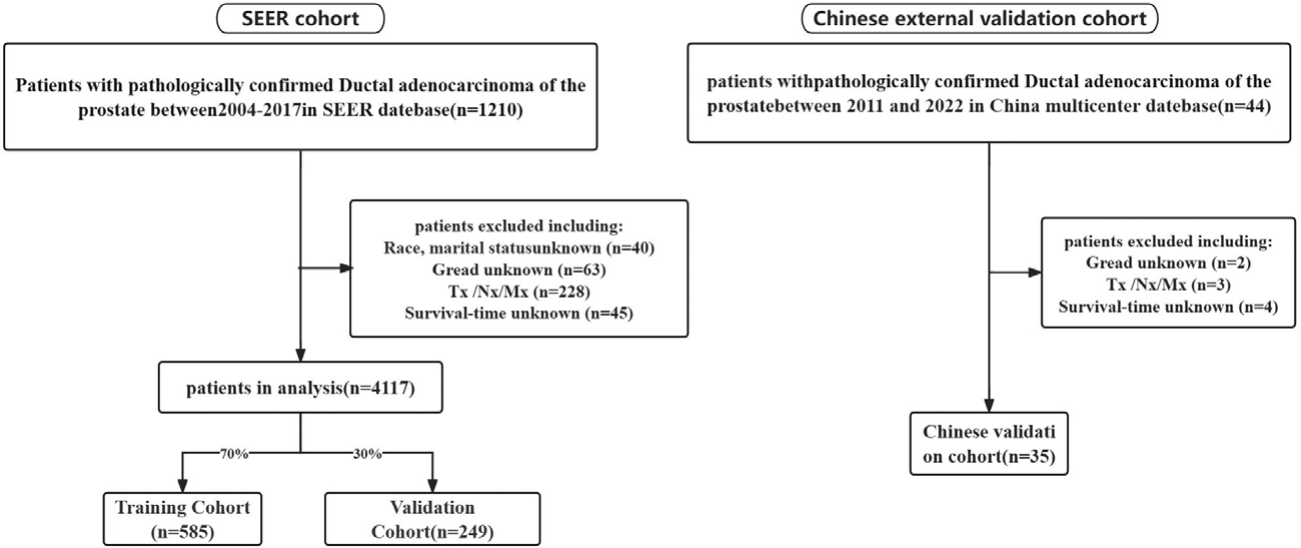
Flow diagram of the selection process for the study.

Using SEER*Stat software (version 8.4.0.1; http://seer.cancer.gov/) to extract and collate data from a database of patients newly diagnosed with DAC between 2004 and 2017, the inclusion criteria for this study were: Patients with primary cancer sites according to the International Classification of Diseases in Oncology (3rd edition) (ICD-O-3) Select C61.9-Prostate and patients with an ICD-O-3 morphology code of 8500/3 (ductal adenocarcinoma); exclusion criteria were as follows: patients with incomplete follow-up information; patients with unknown tumor differentiation grade; patients with unknown TNM stages; patients with unknown marital status and ethnicity; age < 18 years. The external validation set consisted of 35 patients between 2011 and 2022 from our institution. The primary clinical endpoint was OS. The Workflow is shown in Figure 1.

### 2.3. Statistical analysis

Using the “caret” package in the R software, DAC patients were randomly divided 7:3 into a training group and an internal validation group. The training group was used to construct the line graphs (n = 585), the internal validation cohort (n = 249), and the Chinese external validation cohort (n = 35) to verify the ability of the constructed model. Our study transformed continuous variables into categorical variables using X-tile software and restricted cubic spline (RCS) and compared differences in categorical variables using the chi-square or Fisher exact test.

The distributions of demographic and clinical variables in the 2 cohorts were compared using chi-square tests and the median of the interquartile range (IQR) was used to describe continuous variables. Categorical variables were expressed using numbers and percentages. OS was chosen as the study indicator. A 95% confidence interval (95% CI) was also constructed for each independent factor. In the training cohort, we included factors with *P* < .05 in multivariate Cox regression from univariate Cox regression to identify independent prognostic factors. According to the results of multivariate Cox regression, a nomogram was established for predicting the prognosis of patients with DAC. We also verified its validity by receiver operating characteristic (ROC) curve, C-index, calibration plots, DCA and Kaplan–Meier curve analyses. All statistical analyses were performed in R software v4.2.2 (https://www.r-project.org/), Adobe Photoshop 2021. *P* values < 0.05 were considered statistically significant. Due to public availability and anonymized patient information, this study was exempted from obtaining approval from the institutional review board.

## 3. Results

### 3.1. Patients characteristics

**Figure 2.**
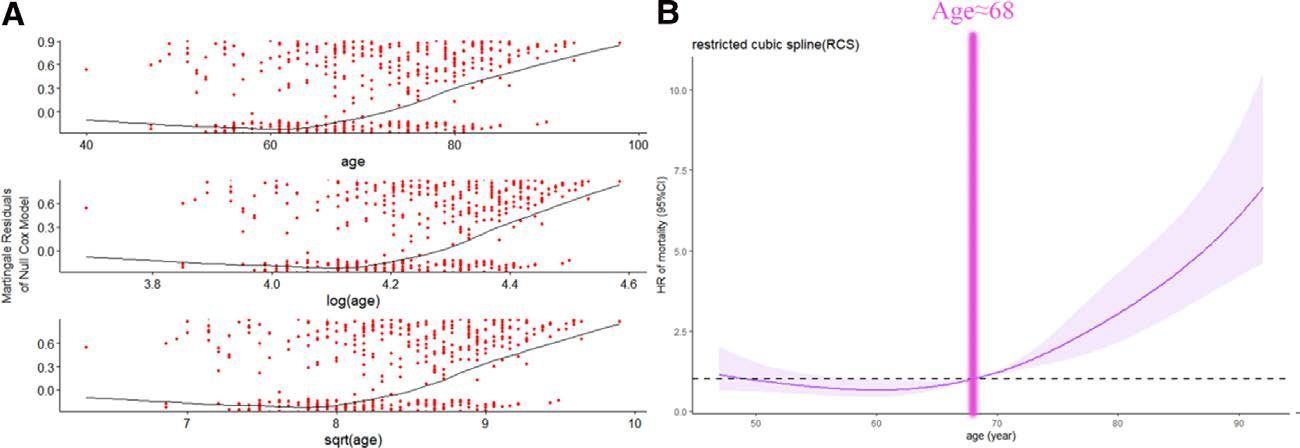
(A) Diagnostic plot of the non-linear relationship between age and prognosis. (B) Restricted cubic spline (RCS) of predicted age and risk of death. Risk ratios are indicated by solid lines and 95% CI by shaded areas.

A total of 834 patients diagnosed with DAC from 2004 to 2017 were identified in our study, and 834 patients were randomly assigned to the training and validation cohorts in a 7:3 ratio, with 585 patients assigned to the training cohort and 249 patients assigned to the validation cohort. The median survival times for the training and validation cohorts were 57 months (IQR: 33, 98 months) and 58 months (IQR: 34, 101 months), respectively, where the median age of all patients was 69 years, with a minimum age of 40 years and a maximum age of 98 years, and a mean age of 69.68 years. Patients were categorized into ≤ 68 years and > 68 years groups based on the results of the RCS (Fig. 2). 50.6% of all patients were diagnosed > 68 years of age and the majority of patients were Caucasian (78.5%) and married in approximately 91% of cases. The Gleason score 6 to 7 was in 39.2% of patients; regarding tumor differentiation grade, I to II and III to IV each accounted for 50%. In terms of treatment, 614 patients (71.4%) underwent surgery, 312 patients (37.4%) had LND, the majority of patients (97.6%) did not receive chemotherapy and approximately 30.6% had radiotherapy. The 1-, 3-, and 5-year OS for patients in the surgical group was 94.1%, 82.6%, and 72.3% (Table 1). In addition, the majority of patients (38.2%) had insignificant PSA elevation, with PSA < 10 ng/mL. There were no significant differences in clinicopathologic characteristics between the training set and the validation set, and the specific clinicopathologic characteristics are shown in Table 2. The Chinese external validation set consisted of 34 patients from our institution (Table 3).

**Table 1:** The OS for patients in the surgical and non-surgical group.

Time (yr)	Surgery patient				Upper 95% CI
	n.risk	n.event	Survival	Lower 95% CI	
1	400	13	0.941	0.919	0.964
3	313	26	0.826	0.79	0.863
5	212	18	0.723	0.679	0.77
	Non-surgical patient				
Time (yr)	n.risk	n.event	Survival	Lower 95% CI	Upper 95% CI
1	149	13	0.92	0.879	0.963
3	105	26	0.753	0.688	0.824
5	72	18	0.607	0.53	0.694

OS = overall survival.

**Table 2:** Univariate and multivariate Cox analyses of clinicopathological characteristics of DAC patients.

Characteristics	Univariate analysis		Multivariate analysis	
	HR (95% CI)	*P* value	HR (95% CI)	*P* value
Marital				
Married	Reference			
Single (never married)	1.46 (0.97–2.2)	.068		
Race				
Black	Reference			
White	0.99 (0.65–1.5)	.951		
Others*	0.98 (0.55–1.77)	.95		
age				
˃68	Reference			
≤68	0.36 (0.27–0.48)	**<.001**	0.46 [0.34, 0.62]	**<.001**
Surgery				
No	Reference			
Yes	0.62 (0.48–0.82)	**.001**	0.44 [0.37, 0.95]	**.017**
Chemotherapy				
No/Unknown	Reference			
Yes	2.07 (0.97–4.4)	.060		
Radiotherapy				
No/Unknown	Reference			
Yes	1.04 (0.78–1.37)	.803		
LND				
No	Reference			
Yes	0.29 (0.21–0.4)	**<.001**	0.45 [0.30, 0.68]	**<.001**
T stage				
T1	Reference			
T2	0.37 (0.27–0.52)	**<.001**	0.52 [0.36, 0.74]	**<.001**
T3	0.32 (0.22–0.47)	**<.001**	0.66 [0.33, 1.35]	.257
T4	1.32 (0.92–1.88)	.129	1.70 [1.03, 2.80]	**.039**
N stage				
N0	Reference			
N1	1.96 (1.34–2.87)	**<.001**	1.87 [1.18, 2.96]	**.008**
M stage				
M0	Reference			
M1	5.71 (4.25–7.66)	**<.001**	5.05 [2.95, 8.63]	**<.001**
Pathologic stage				
I–II	Reference			
III–IV	1.58 (1.21–2.06)	**.001**	0.72 [0.40, 1.27]	.257
Gleason score				
Gleason score 5–6	Reference			
Gleason score 7–8	3.05 (0.74–12.58)	.123	3.21 [0.74, 13.87]	.119
Gleason score 9–10	9.3 (2.24–38.64)	**.002**	6.40 [1.47, 27.91]	**.013**
Unknown	6.81 (1.69–27.54)	**.007**	4.38 [1.03, 18.59]	**.045**
PSA				
<10	Reference			
10–50	1.64 (1.06–2.54)	**.028**	1.71 [1.07, 2.75]	**.025**
≥50	3.37 (1.92–5.94)	**<.001**	1.04 [0.55, 1.99]	.904
Unknown	1.87 (1.33–2.61)	**<.001**	1.56 [1.01, 2.41]	**.044**

* Others: American Indian, AK Native, Asian and Pacific Islander.

DAC = ductal adenocarcinoma of the prostate, HR = hazard ratio, LND = lymph node dissection.

*P* < 0.05 considered statistically significant.

**Table 3:** Baseline demographic and clinical characteristics in the Chinese Validation cohort.

Characteristic	Chinese validation cohort (N = 35)	Characteristic	Chinese validation cohort (N = 35)
Marital, n (%)		N stage, n (%)	
Married	35 (100%)	N0	31 (88.6%)
Single (never married)	0	N1	4 (11.4%)
age, n (%)		M stage, n (%)	
˃68	20 (57.1%)	M0	30 (85.7%)
≤68	15 (42.8%)	M1	5 (14.3%)
Surgery, n (%)		Pathologic stage, n (%)	
No	7 (20%)	I-II	14 (16.3%)
Yes	28 (80%)	III-IV	21 (13.5%)
Chemotherapy, n (%)		Gleason score, n (%)	
No	20 (57.1%)	Gleason score 5–6	2 (5.7%)
Yes	15 (34.3%)	Gleason score 7–8	19 (54.2%)
Radiotherapy, n (%)		Gleason score 9–10	8 (22.9%)
No	28 (80%)	Unknown	6 (17.1%)
Yes	7 (20%)	PSA, n (%)	
LND, n (%)		˂10	14 (40%)
No	24 (68.5%)	≥50	5 (14.3%)
Yes	11 (31.4%)	10–50	13 (37.1%)
T stage, n (%)		Unknown	3 (8.6%)
T1	9 (25.7%)		
T2	9 (25.7%)		
T3	14 (40%)		
T4	3 (8.5%)		

LND = lymph node dissection.

### 3.2. Univariate and multivariate Cox regression analyses

Table 3 summarizes the results of univariate and multivariate Cox regression analysis of the training cohort. Univariate and multivariate Cox analysis indicated that age at diagnosis (*P* < .001), surgery (*P* = .017), LND (*P* < .001), T-stage (*P* < .001), N-stage (*P* = .008), M-stage (*P* < .001), Gleason score (*P* = .013), PSA (*P* = .025) were prognostic factors for patients with ductal adenocarcinoma of the prostate. Tumor differentiation grade did not appear to be an independent prognostic factor in the multivariate Cox regression, contrary to the results of the univariate Cox regression. Using hazard ratios and 95% CI to show the independent effects of predictors in patients with DAC (Table 4).

**Table 4:** Univariate and multivariate Cox analyses of clinicopathological characteristics of DAC patients.

Characteristics	Univariate analysis		Multivariate analysis	
	HR (95% CI)	*P* value	HR (95% CI)	*P* value
Marital				
Married	Reference			
Single (never married)	1.46 (0.97–2.2)	.068		
Race				
Black	Reference			
White	0.99 (0.65–1.5)	.951		
Others*	0.98 (0.55–1.77)	.95		
age				
˃68	Reference			
≤68	0.36 (0.27–0.48)	**<.001**	0.46 (0.34, 0.62)	**<.001**
Surgery				
No	Reference			
Yes	0.62 (0.48–0.82)	**.001**	0.44 (0.37, 0.95)	**.017**
Chemotherapy				
No/Unknown	Reference			
Yes	2.07 (0.97–4.4)	.06		
Radiotherapy				
No/Unknown	Reference			
Yes	1.04 (0.78–1.37)	.803		
LND				
No	Reference			
Yes	0.29 (0.21–0.4)	**<.001**	0.45 (0.30, 0.68)	**<.001**
T stage				
T1	Reference			
T2	0.37 (0.27–0.52)	**<.001**	0.52 (0.36, 0.74)	**<.001**
T3	0.32 (0.22–0.47)	**<.001**	0.66 (0.33, 1.35)	.257
T4	1.32 (0.92–1.88)	.129	1.70 (1.03, 2.80)	**.039**
N stage				
N0	Reference			
N1	1.96 (1.34–2.87)	**<.001**	1.87 (1.18, 2.96)	**.008**
M stage				
M0	Reference			
M1	5.71 (4.25–7.66)	**<.001**	5.05 (2.95, 8.63)	**<.001**
Pathologic stage				
I–II	Reference			
III–IV	1.58 (1.21–2.06)	**.001**	0.72 (0.40, 1.27)	.257
Gleason score				
Gleason score 5–6	Reference			
Gleason score 7–8	3.05 (0.74–12.58)	.123	3.21 (0.74, 13.87)	.119
Gleason score 9–10	9.3 (2.24–38.64)	**.002**	6.40 (1.47, 27.91)	**.013**
Unknown	6.81 (1.69–27.54)	**.007**	4.38 (1.03, 18.59)	**.045**
PSA				
<10	Reference			
10–50	1.64 (1.06–2.54)	**.028**	1.71 (1.07, 2.75)	**.025**
≥50	3.37 (1.92–5.94)	**<.001**	1.04 (0.55, 1.99)	.904
Unknown	1.87 (1.33–2.61)	**<.001**	1.56 (1.01, 2.41)	**.044**

* Others: American Indian, AK Native, Asian and Pacific Islander.

DAC = ductal adenocarcinoma of the prostate, HR = hazard ratio, LND = lymph node dissection.

*P* < 0.05 considered statistically significant.

### 3.3. Survival analysis

**Figure 3.**
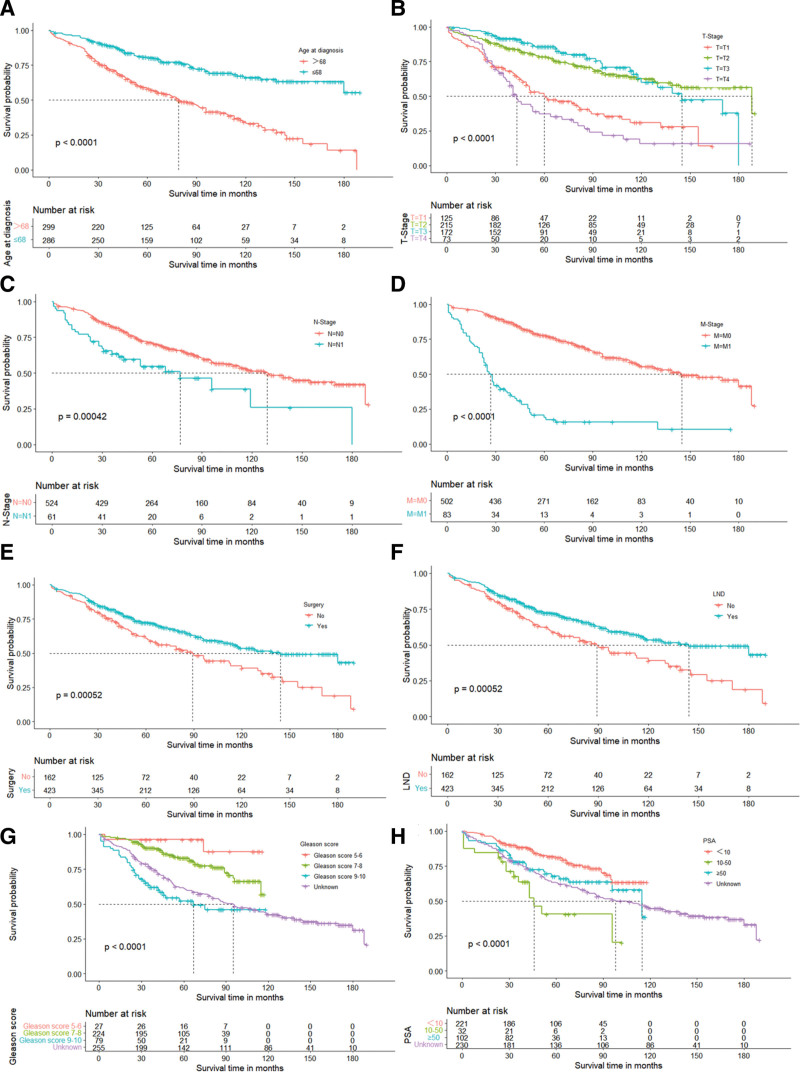
K-M survival analysis of OS for DAC patients according to different independent prognostic factors. K-M survival analysis for DAC patients with (A) Age, (B) T-stage, (C) N-stage, (D) M-stage, (E) surgical, (F) lymph node dissection (LND), (G) Gleason score and (H) PSA. DAC = ductal adenocarcinoma of the prostate, OS = overall survival.

The 8 independent prognostic factors identified were included in the Kaplan–Meier survival analysis, and differences between curves were assessed using the log-rank test for OS. The Kaplan–Meier survival curves showed that patients older than 68 years had a worse prognosis (*P* < .0001) (Fig. 3A). In addition, patients had relatively poorer OS with increasing levels of T-stage (*P* < .001), N-stage (*P* < .0001), and M-stage (*P* < .0001) (Fig. 3B–D). Patients who underwent surgery or LND tended to have higher survival (*P* < .001) (Fig. 3E and F). Patients with higher Gleason scores had lower OS (*P* < .001) (Fig. 3G). Among PSA levels, the prognosis was relatively poor when patients had PSA = 10 to 50 ng/mL (*P* < .001) (Fig. 3H). These findings also confirm the results of the COX regression analysis described above.

### 3.4. Development of the nomogram

**Figure 4.**
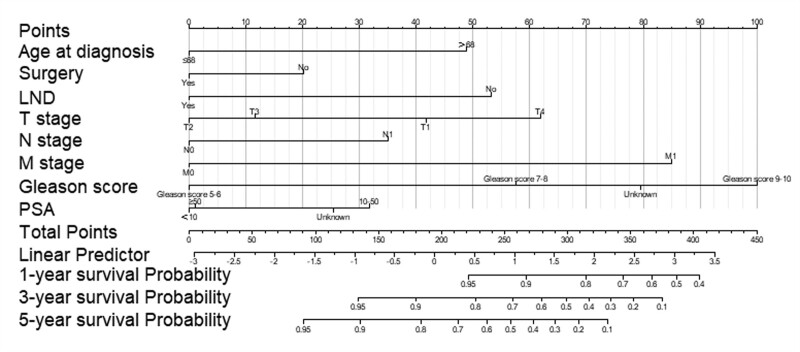
The nomogram to predict OS at 1-, 3-, 5-yr for patients with DAC. DAC = ductal adenocarcinoma of the prostate, LND = lymph node dissection, OS = overall survival.

Based on the results of the univariate and multifactorial COX regression risk models, we developed a newly constructed nomogram that can individually predict survival prognosis at 1, 3, and 5 years in patients with ductal adenocarcinoma of the prostate (Fig. 4). The length of each variable in the nomogram indicates the magnitude of the effect of that factor on the survival prognosis of the patient. Nomogram scores for each prognostic variable are shown in Supplementary Fig 1, http://links.lww.com/MD/L287.

### 3.5. Validation of the nomogram

**Figure 5.**
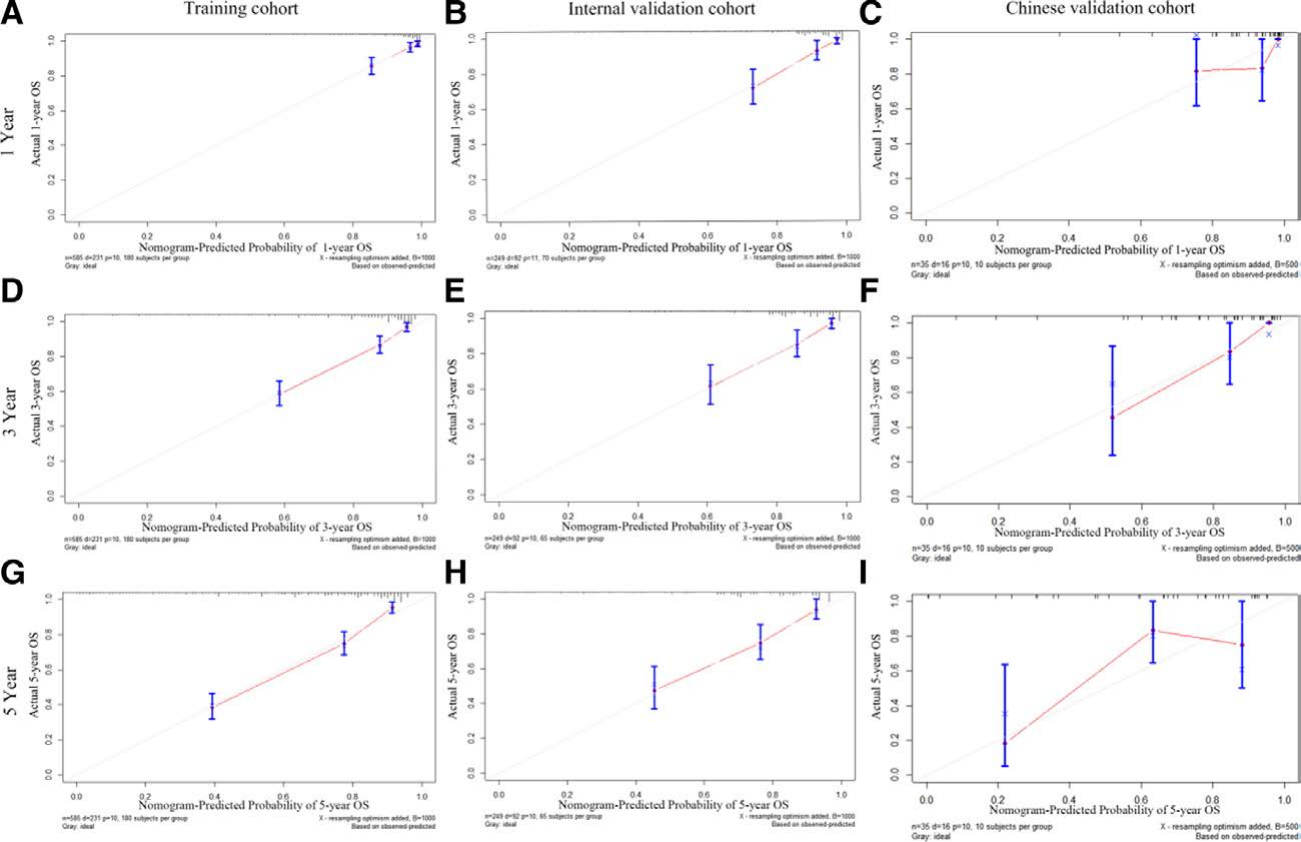
Calibration plots of OS nomogram model. 1, 3, 5 yr calibration plot of OS using the SEER training cohort (A, D, G); 1, 3, 5 yr calibration plot of OS using the SEER internal validation cohort (B, E, H); 1, 3, 5 yr calibration plot of OS using the Chinese validation cohort (C, F, I). OS = overall survival, SEER = Surveillance, Epidemiology, and End Results.

The excellent recognition ability of the nomogram was verified using the SEER internal validation cohort and the Chinese external validation cohort. The C-index of the nomogram in the training cohort was 0.767 (95% CI = 0.716–0.818). In both validation cohorts, the C-index for the internal validation cohort was 0.786 (95% CI = 0.755–0.817); the C-index for the Chinese external validation cohort was 0.817 (95% CI = 0.773–0.901), which was >0.7 in both cases (Table 5). Figure 5 shows the calibration plots between predictions and actual observations for the 1-, 3-, 5-year OS in the training cohort (Fig. 5A, D, G), validation cohort (Fig. 5B, E, H) and Chinese external verification cohort (Fig. 5C, F, I) of the nomogram predictions, and the results show that the nomogram has good calibration capability.

**Table 5:** The C-indexes for predictions of OS.

Group	OS	
	C-index	95% CI
Training cohort	0.767	0.716–0.818
SEER internal validation cohort	0.786	0.755–0.817
Chinese external validation cohort	0.817	0.773–0.901

OS = overall survival, SEER = Surveillance, Epidemiology, and End Results.

**Figure 6.**
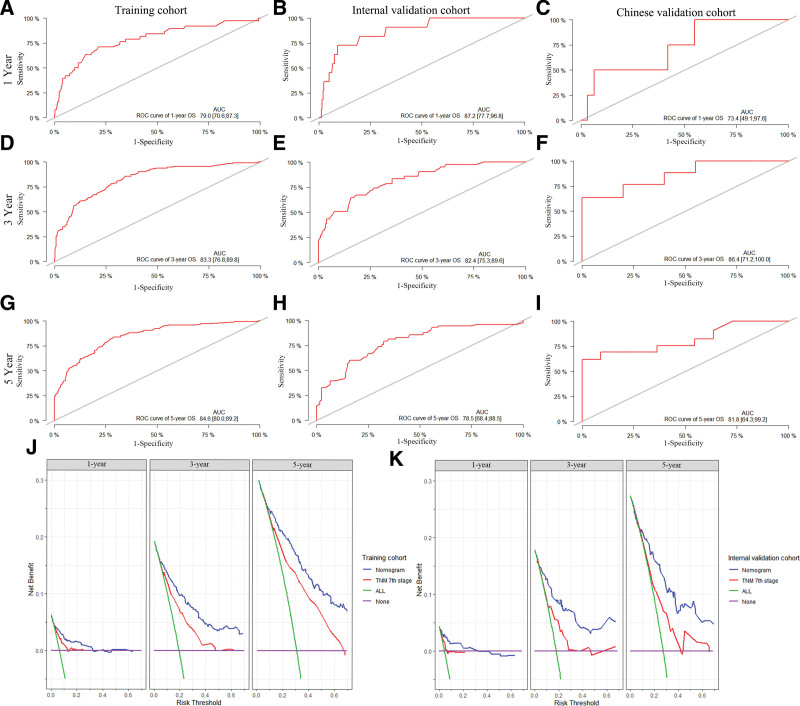
The AUCs of the nomograms that predicted 1-, 3- and 5-yr overall survival of ADC in the training cohort (A, D, G). Similar superiority in nomogram prediction accuracy was also observed in the SEER internal validation cohort (B, E, H) and the Chinese validation cohort (C, F, I). DCA curves for 1, 3, and 5 yr in the training cohort (J); DCA curves for 1-, 3-, 5-yr in the validation cohort (K). AUCs = the area under the curve, DCA = decision curve analysis, SEER = Surveillance, Epidemiology, and End Results.

In addition, we also evaluated the specificity and sensitivity of the nomogram by drawing ROC curves. The area under the ROC curves (AUC) in the training cohort showed good accuracy in predicting the risk of death at 1, 3, and 5 years (1-year: 0.790; 3-year: 0.833; 5-year: 0.846) (Fig. 6A, D, G). Meanwhile, the results were similar in the validation cohort (1-year: 0.872; 3-year: 0.824; 5-year: 0.785) (Fig. 6B, E, H) and Chinese external validation cohort (1-year: 0.734; 3-year: 0.864; 5-year:0.818) (Fig. 6C, F, I). And the DCA was applied to compare the applicability and clinical decision making of the nomogram with the AJCC TNM stages system. The results showed that the nomogram was more capable of predicting OS than the AJCC 7 stage. Based on these results, the model proved to have reliable predictive ability (Fig. 6J and K).

### 3.6. Establishment of a stratified risk system based on the nomogram

**Figure 7.**
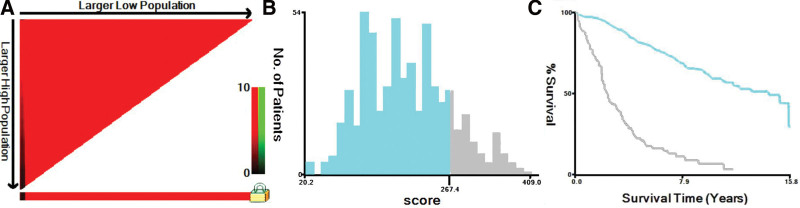
The X-tile analysis of best-cutoff points of risk stratification. (A) X-tile plot of training sets in risk stratification; (B) the cutoff point was highlighted using a histogram of the entire cohort; (C) the distinct prognosis determined by the cutoff point was shown using a Kaplan–Meier plot (low subset = blue, high subset = gray).

**Figure 8.**
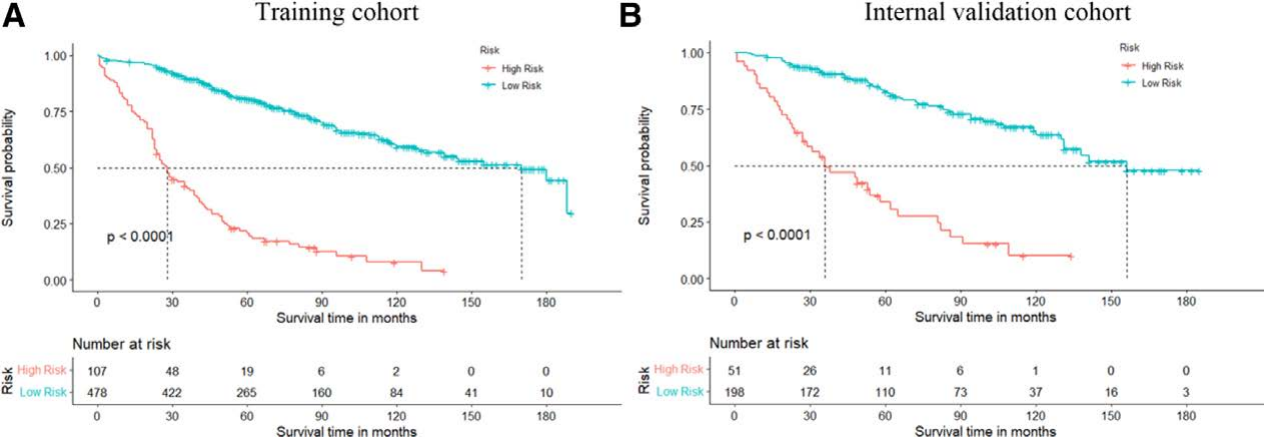
K-M survival curves of patients with DAC in the high-risk and low-risk groups. (A) K-M survival curve of patients with DAC in the high-risk and low-risk groups in the training cohort; (B) K-M survival curve of patients with DAC in the high-risk and low-risk groups in the validation cohort.

In addition, we calculated each patient score based on the prognostic score generated from the nomogram, using the X-tile (3.6.1 software 20, http://medicine.yale.edu/lab/rimm/research/software.aspx) to select the best cutoff value (Fig. 7). Patients were divided into a high-risk group (total score > 267.4) and a low-risk group (total score ≤ 267.4) to further test the feasibility and validity of the prediction model. K-M survival analysis showed a significant difference in prognosis between the high-risk and low-risk groups (*P* < .001), and patients in the high-risk group had a significantly lower OS than individuals in the low-risk group, indicating that the model could well identify individuals at high risk of cancer in terms of OS (Fig. 8).

### 3.7. Construction of online dynamic nomogram

Finally, to facilitate the clinical application of the nomogram, we further generated a web-based dynamic nomogram. People can easily input values of the 8 predictors followed by a click of the “Predict” button, then the survival probability and 95% confidence interval are exported on the right side of the interface (dynamic nomogram: https://lc123456.shinyapps.io/DAC-Nomogram/).

## 4. Discussion

DAC is a rare and aggressive sub-type of PCa, and patients with DAC tend to metastasize more distantly and have a higher mortality rate than those with conventional alveolar prostate carcinoma.^[16]^ DAC with other prostate carcinoma sub-types is more common than pure DAC.^[17]^ Because DAC often originates from the prostatic ducts around the primary urethra, most patients are found in the clinic with urinary obstruction or hematuria.^[18]^ There is evidence that DAC has a lower PSA value at the time of diagnosis compared to APC and is therefore diagnosed at a more advanced stage.^[19]^ Our study also confirmed this, with the majority of patients having a PSA < 10 ng/mL. In addition, although DAC often occurs in combination with other prostate carcinoma sub-types, one study found no significant difference in OS between mixed and pure DPC.^[20,21]^Multiple institutional series have consistently demonstrated worse pathological findings for patients with DAC than for those with PAC, with higher rates of Gleason score ≥ 7 disease (ISUP Grade Group ≥ 4).^[22–24]^ Our analysis using a large sample of cases from the SEER database also found poor pathological outcomes in DAC patients, with a Gleason score ≥ 7 in 51.9% of patients. Patients with DAC have a higher stage of pathology, which may be a contributing factor to the low survival rate.

Nowadays, although there are no official guidelines, treatment options for DAC are usually the same as for adenocarcinoma of prostate. Surgical treatment (robotic and otherwise) is preferred for patients with eradicable disease and a life expectancy of more than 10 years while definitive EBRT or brachytherapy is usually chosen for locally advanced disease and ADT alone for patients with metastatic disease or a shortened life expectancy.^[25]^ Ranasinghe et al^[26]^ retrospectively included patients with DAC treated with surgery and patients with APC treated with surgery, and they reported a 5-year OS of 88% for DPC in the surgical group compared with 97% for APC, with poorer OS and metastasis-free survival in patients with DPC. Our study design did not include a direct comparison between patients with DPC and patients with adenoidal blasts. However, our study found a 5-year OS of 72.3% in patients with surgically treated ductal adenocarcinoma of the prostate. Therefore, in this setting, we may hypothesize an overlap in the 5-year survival rates for DPC between the 2 studies. Indirectly, such findings may confirm that the ductal pattern has a worse prognosis than typical PCa. Overall, the current evidence demonstrates that men undergoing radical prostatectomy for DAC have a worse prognosis than men with high-risk PAC. In addition, Bergamin et al^[27]^ found that patients with DCA did not respond as well to radiotherapy as patients with APC. In our study, although 30.6% of patients received radiotherapy, this did not seem to affect the long-term survival of patients. Nowadays, preoperative neoadjuvant therapy for some cancers can now reduce postoperative adverse effects and improve cancer survival rates. However, a neoadjuvant study reported no measurable benefit of ADT in the treatment of DAC, and none of the patients involved achieved a reduction in pathologic stage. Even with combination therapy, only 10% of male patients with DAC treated with neoadjuvant ADT plus abiraterone or ADT plus docetaxel achieved complete pathologic remission, and this remission did not translate into an OS benefit.^[28]^ This may be related to the early resistance of DAC to ADT. A systematic review has shown that multimodality therapy combined with radical prostatectomy, systemic therapy, and radiotherapy improves prognosis in patients with high-risk PAC and poor pathology.^[29]^ Multimodality therapy combines multiple levels of treatment, including a combination of surgery and neoadjuvant, adjuvant, or salvage therapy, and is often used in high-risk cancers to improve long-term outcomes by reducing local tumor load and eradicating micrometastatic disease, with the ultimate goal of improving long-term outcomes.^[30]^

Previously, some studies subjectively classified patients into different age groups, which may have led to statistical bias.^[31]^ To address this issue, we applied the RCS to define the optimal cutoff value for age, based on survival status and survival time. Our study showed that older age (especially > 68 years) is a risk factor for the prognosis of patients with DAC. In terms of treatment, most patients underwent surgery and a relatively low proportion received radiotherapy and chemotherapy. Univariate and multivariate COX analyses showed that patients with higher AJCC TNM stages and higher Gleason scores tended to have a worse prognosis. In addition, univariate COX analysis indicated that grade of pathological differentiation was a prognostic factor, but the results of multivariate COX analysis indicated that grade of pathological differentiation did not have a higher prognostic value for DAC. This may be related to the higher grade of pathological differentiation in most DAC patients. Finally, we identified 8 variables as important predictors of patient prognosis. The AUC, and DCA values of the nomogram were higher than those of the AJCC7 system, suggesting its superior discrimination ability and clinical significance. When using the nomogram to calculate the OS of DAC, it also showed a high C-index and AUC, demonstrating that the nomogram has high accuracy and practicability in predicting prognosis. In addition, this study categorized patients into high-risk and low-risk groups for OS probability. KM analysis showed a statistically significant difference in survival time between these 2 groups (*P* < .0001). Many influencing factors were evaluated in this study and accurate predictions can be made based on all the important factors included in the nomogram. The creation of nomogram will help to differentiate between patients at high and low risk and thus design personalized treatments for patients with DCA. Therefore, if the nomogram reveals that a patient score belongs to the high-risk group, clinicians can focus on that patient and make timely adjustments to the subsequent treatment, for example, by implementing combined-modality therapy as soon as possible, in order to help the patient achieve a better prognosis. This study had several limitations. First, we were unable to obtain more information from the SEER database, such as endocrine therapy, immune therapy, laboratory tests and chemotherapy regimens, which may have led to some bias. In addition, the retrospectively collected data may have led to certain selection biases that could not be fully overcome by statistical analysis. In the future, we will combine multiple centers to incorporate additional variables, such as androgen deprivation therapy (ADT), chemotherapy regimen, cycle, and dose, and we plan to augment the model with additional repositories in various countries during subsequent analyses. In order to validate the feasibility of the model at more organizations, our next step will be to develop a downloadable web application to be shared by more organizations.

## 5. Conclusion

In this study, age, T-stage, N-stage, M-stage, surgery, LND, Gleason score and PSA were identified as independent prognostic factors for DAC patients, and a new nomogram was constructed to predict 1-, 3-, 5-year OS for DAC patients.

## Author contributions

**Conceptualization:** Yinglei Wang.

**Data curation:** Cheng Li, Guangming Shan.

**Project administration:** Cheng Li.

**Resources:** Zhengqiang Wan, Baoquan Yang.

**Software:** Guangming Shan.

**Validation:** Zhengqiang Wan.

**Writing – original draft:** Cheng Li.

**Writing – review & editing:** Yinglei Wang.

## Supplementary Material

## References

[1] Mattiuzzi C Lippi G. Current cancer epidemiology. J Epidemiol Glob Health. 2019;9:217–22. 31854162 10.2991/jegh.k.191008.001 PMC7310786

[2] Jemal A Center MM DeSantis C. Global patterns of cancer incidence and mortality rates and trends. Cancer Epidemiol Biomarkers Prev. 2010;19:1893–907. 20647400 10.1158/1055-9965.EPI-10-0437

[3] Benafif S Eeles R. Genetic predisposition to prostate cancer. Br Med Bull. 2016;120:75–89. 27941040 10.1093/bmb/ldw039

[4] Rhoden EL Averbeck MA. [Prostate carcinoma and testosterone: risks and controversies]. Arq Bras Endocrinol Metabol. 2009;53:956–62. 20126847 10.1590/s0004-27302009000800008

[5] Humphrey PA Moch H Cubilla AL. 2016 WHO Classification of tumours of the urinary system and male genital organs-part B: prostate and bladder tumours. Eur Urol. 2016;70:106–19. 26996659 10.1016/j.eururo.2016.02.028

[6] Sung H Ferlay J Siegel RL. Global Cancer Statistics 2020: GLOBOCAN estimates of incidence and mortality worldwide for 36 Cancers in 185 Countries. CA Cancer J Clin. 2021;71:209–49. 33538338 10.3322/caac.21660

[7] Melicow MM Pachter MR. Endometrial carcinoma of prostatic utricle (uterus masculinus). Cancer. 1967;20:1715–22. 4168340 10.1002/1097-0142(196710)20:10<1715::aid-cncr2820201022>3.0.co;2-e

[8] Orihuela E Green JM. Ductal prostate cancer: contemporary management and outcomes. Urol Oncol. 2008;26:368–71. 18367098 10.1016/j.urolonc.2007.05.028

[9] Hertel JD Humphrey PA. Ductal adenocarcinoma of the prostate. J Urol. 2011;186:277–8. 21600616 10.1016/j.juro.2011.04.031

[10] Meeks JJ Zhao LC Cashy J. Incidence and outcomes of ductal carcinoma of the prostate in the USA: analysis of data from the surveillance, epidemiology, and end results program. BJU Int. 2012;109:831–4. 21883856 10.1111/j.1464-410X.2011.10520.x

[11] Igdem S Spiegel DY Efstathiou J. Prostatic duct adenocarcinoma: clinical characteristics, treatment options, and outcomes – a Rare Cancer Network study. Onkologie. 2010;33:169–73. 20389142 10.1159/000288710

[12] Morgan TM Welty CJ Vakar-Lopez F. Ductal adenocarcinoma of the prostate: increased mortality risk and decreased serum prostate specific antigen. J Urol. 2010;184:2303–7. 20952027 10.1016/j.juro.2010.08.017 PMC3111052

[13] Hiramatsu K Tsuzaka Y Kaneko T. [Ductal adenocarcinoma of the prostate: a report of 7 cases]. Nihon Hinyokika Gakkai Zasshi. 2012;103:671–4. 23342927 10.5980/jpnjurol.103.671

[14] Xia PF Zhang EH Li XG. Report of two cases of ductal adenocarcinoma of the prostate and review of the literature. Chinese J Male Sci. 2022;28:183–5.

[15] Chao L. Report of 45 cases of ductal adenocarcinoma of the prostate and review of the literature. Chin J Male Sci. 2022;28:129–34. 37462484

[16] Bell KJ Del MC Wright G. Prevalence of incidental prostate cancer: a systematic review of autopsy studies. Int J Cancer. 2015;137:1749–57. 25821151 10.1002/ijc.29538 PMC4682465

[17] Zhou M. High-grade prostatic intraepithelial neoplasia, PIN-like carcinoma, ductal carcinoma, and intraductal carcinoma of the prostate. Mod Pathol. 2018;31:S71–79. 29297491 10.1038/modpathol.2017.138

[18] Cozzi S Bardoscia L Najafi M. Ductal prostate cancer: clinical features and outcomes from a multicenter retrospective analysis and overview of the current literature. Curr Urol. 2022;16:218–26. 36714233 10.1097/CU9.0000000000000118 PMC9875213

[19] Knipper S Preisser F Mazzone E. Contemporary comparison of clinicopathologic characteristics and survival outcomes of prostate ductal carcinoma and acinar adenocarcinoma: a population-based study. Clin Genitourin Cancer. 2019;17:231–237.e2. 31080021 10.1016/j.clgc.2019.04.009

[20] Amin A Epstein JI. Pathologic stage of prostatic ductal adenocarcinoma at radical prostatectomy: effect of percentage of the ductal component and associated grade of acinar adenocarcinoma. Am J Surg Pathol. 2011;35:615–9. 21383610 10.1097/PAS.0b013e31820eb25b PMC4425125

[21] Epstein JI Egevad L Amin MB. The 2014 International Society of Urological Pathology (ISUP) Consensus Conference on Gleason grading of prostatic carcinoma: definition of grading patterns and proposal for a new grading system. Am J Surg Pathol. 2016;40:244–52. 26492179 10.1097/PAS.0000000000000530

[22] Jang WS Shin SJ Yoon CY. Prognostic significance of the proportion of ductal component in ductal adenocarcinoma of the prostate. J Urol. 2017;197:1048–53. 27916712 10.1016/j.juro.2016.11.104

[23] Harkin T Elhage O Chandra A. High ductal proportion predicts biochemical recurrence in prostatic ductal adenocarcinoma. BJU Int. 2019;124:907–9. 31136054 10.1111/bju.14831

[24] Kim A Kwon T You D. Clinicopathological features of prostate ductal carcinoma: matching analysis and comparison with prostate acinar carcinoma. J Korean Med Sci. 2015;30:385–9. 25829805 10.3346/jkms.2015.30.4.385 PMC4366958

[25] Ranasinha N Omer A Philippou Y. Ductal adenocarcinoma of the prostate: a systematic review and meta-analysis of incidence, presentation, prognosis, and management. BJUI Compass. 2021;2:13–23. 35474657 10.1002/bco2.60 PMC8988764

[26] Ranasinghe W Shapiro DD Hwang H. Ductal prostate cancers demonstrate poor outcomes with conventional therapies. Eur Urol. 2021;79:298–306. 33279304 10.1016/j.eururo.2020.11.015

[27] Bergamin S Eade T Kneebone A. Ductal carcinoma of the prostate: an uncommon entity with atypical behaviour. Clin Oncol (R Coll Radiol). 2019;31:108–14. 30471806 10.1016/j.clon.2018.10.011

[28] Ranasinghe W Brooks NA Elsheshtawi MA. Patterns of metastases of prostatic ductal adenocarcinoma. Cancer. 2020;126:3667–73. 32453443 10.1002/cncr.32957

[29] Tosco L Briganti A D’Amico AV. Systematic review of systemic therapies and therapeutic combinations with local treatments for high-risk localized prostate cancer. Eur Urol. 2019;75:44–60. 30286948 10.1016/j.eururo.2018.07.027

[30] Maithel SK D’Angelica MI. An update on randomized clinical trials in advanced and metastatic colorectal carcinoma. Surg Oncol Clin N Am. 2010;19:163–81. 19914565 10.1016/j.soc.2009.09.013

[31] Huang G Zhang H Shi H. Clinicopathological and immunological profiles of prostate adenocarcinoma and neuroendocrine prostate cancer. World J Surg Oncol. 2022;20:407. 36572885 10.1186/s12957-022-02841-6 PMC9793563

