# Supplementary figures and images for: Construction and validation of a prognostic nomogram for ductal adenocarcinoma of the prostate: A population-based study
Source: Medicine (Baltimore). 2024 Jan 12;103(2):e36877. doi: 10.1097/MD.0000000000036877 (PMC10783338; doi:10.1097/MD.0000000000036877)

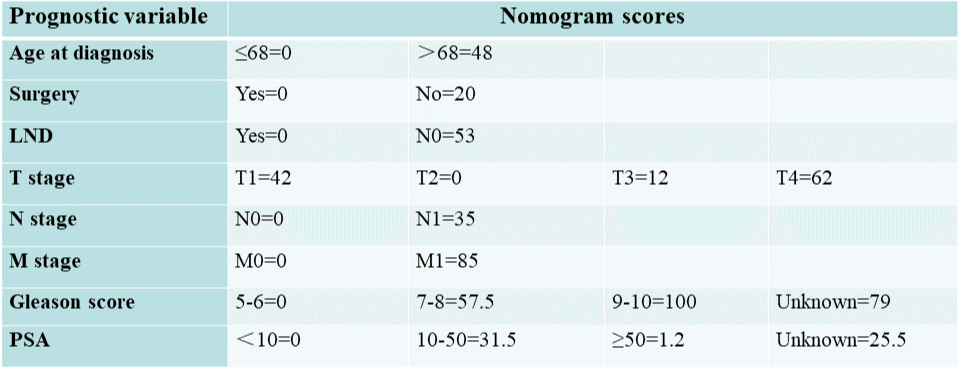

Supplement: Supplementary file 1 [file medi-103-e36877-s001.tiff]
